# Labor analgesia: knowledge, attitudes, and practices among pregnant women and husbands in Wuhan: a cross-sectional study

**DOI:** 10.1080/07853890.2026.2670069

**Published:** 2026-05-12

**Authors:** Li Wang, Yuanyuan Wu, Zemei Mao, Yu Yang

**Affiliations:** ^a^ Department of Anesthesiology, Wuhan Children’s Hospital (Wuhan Maternal and Child Healthcare Hospital), Tongji Medical College, Huazhong University of Science & Technology, Wuhan, China; ^b^ Department of Gynecology and Obstetrics, Wuhan Children’s Hospital (Wuhan Maternal and Child Healthcare Hospital), Tongji Medical College, Huazhong University of Science & Technology, Wuhan, China

**Keywords:** Labor analgesia, pregnancy, knowledge, attitudes, practices

## Abstract

**Background:**

Despite advancements in labor analgesia, its adoption in China remains suboptimal. This study evaluated the knowledge, attitudes, and practices (KAP) of pregnant women and their husbands in Wuhan regarding labor analgesia.

**Materials and methods:**

A cross-sectional survey was conducted using a structured questionnaire to collect demographic information and assess KAP. Correlation analysis, logistic regression, structural equation modeling (SEM), and mediation analyses were performed to evaluate associations and pathways.

**Results:**

Data from 549 couples were analyzed. The KAP scores of pregnant women were 7.19 ± 4.32, 45.66 ± 5.91, and 11.94 ± 3.96. The mean spouse score was 21.69 ± 3.78. Multivariate logistic regression showed that knowledge (OR = 1.347, 95% CI: 1.213–1.497), spousal leadership occupations (OR = 6.211, 95% CI: 1.098–35.125), and commercial/service work (OR = 8.526, 95% CI: 1.264–57.523, all *p* < 0.05) were positively associated with practices in women. SEM and mediation analysis revealed significant direct effects of knowledge on attitude (β = 0.473) and practice (β = 0.544), and of spouse scores on KAP (β = 0.186–0.326, all *p* < 0.05). Spouse scores had indirect effects on attitude (β = 0.154) and practice (β = 0.198, both *p* < 0.05).

**Conclusion:**

Pregnant women in Wuhan showed poor knowledge and practice but positive attitudes towards labor analgesia. Husbands had good knowledge/willingness/involvement. Enhanced education for both is essential to improve uptake.

## KEY MESSAGES

Pregnant women in Wuhan demonstrated poor knowledge and practice but held positive attitudes towards labor analgesia, indicating a significant gap between their willingness to accept and their understanding and use of the procedure.

The awareness and support of husbands played a significant and multifaceted role, having both direct and indirect positive effects on the knowledge, attitudes, and practices of pregnant women regarding labor analgesia.

Enhancing educational interventions targeted at both pregnant women and their spouses is essential to bridge the knowledge-practice gap and improve the uptake of labor analgesia services.

## Introduction

Labor analgesia, commonly referred to in public discussions as ‘painless childbirth’, encompasses a range of techniques to alleviate or eliminate the pain associated with delivery [1]. Labor analgesia, especially epidural analgesia, does not completely remove pain in most cases; rather, it reduces labor pain to a level that is generally acceptable to parturients. Labor pain management comprises pharmacological (e.g. neuraxial analgesia) and non-pharmacological (e.g. doula support, hydrotherapy) approaches [1]. Neuraxial analgesia, particularly epidural analgesia, represents the gold standard due to its demonstrated efficacy and safety [2]. Robust clinical evidence confirms its benefits, indicating that epidural analgesia does not increase the rate of cesarean delivery and may be associated with a modest rise in instrumental delivery, which is generally not clinically significant, without compromising maternal or neonatal outcomes when properly administered [3]. High adoption rates of labor analgesia have been documented in developed nations, with about 60% of parturients in the United States and 30% in the United Kingdom undergoing the management [4]. In contrast, the overall prevalence of labor analgesia in Shanghai (China) remains limited at an estimated 10% [5]. Significant regional disparities exist, with utilization rates ranging from 1.02% in northwest China to 30.77% in eastern regions [6]. Another critical constraint is the anesthesiologist shortage, with an estimation of 0.4 per 10,000 population [7]. In response to these issues, the Chinese government launched the Pilot Program for Labor Analgesia in 2018, identifying 913 hospitals, including several in Wuhan, to promote standardized pain relief during childbirth [8].

Notably, the adoption of labor analgesia in China is significantly influenced by joint decision-making within the family, especially between pregnant women and their husbands, particularly the knowledge, attitudes, and practices (KAP) of both pregnant women and their husbands [9,10]. Adequate knowledge is fundamental in fostering positive attitudes and informed decisions regarding labor analgesia [11]. Besides, positive perceptions of labor analgesia are linked to reduced maternal anxiety, higher birth satisfaction, and improved postpartum mental health [3,12]. However, a qualitative study in China highlighted the limited provider-patient interaction time, thus restricting meaningful discussions on birth pain relief [13]. The knowledge gap is further exacerbated among husbands, who often lack access to structured education on epidural analgesia [14]. Moreover, the adoption of labor analgesia is strongly influenced by health-seeking behaviors, such as regular prenatal class attendance and active discussion of pain relief options with providers [15]. Particularly impactful is spousal involvement, where husbands’ participation in antenatal visits and advocacy during labor can increase utilization rates [16]. These observations highlight the importance of family-centered approaches in promoting effective labor analgesia.

The KAP model has been extensively utilized in public health research to examine relationships between health knowledge, perceptions, and behaviors. While previous studies have employed this framework to investigate women’s views on labor analgesia, three critical limitations emerge [17–19]. For example, Hosseinzadeh et al. used the KAP framework to assess maternal knowledge and attitudes regarding painless labor in a referral hospital in Northern Iran, but their survey was limited to pregnant women and did not evaluate husbands’ knowledge or participation in decision-making [17]. Nabwy Helmi et al. and Paul et al. likewise examined antenatal women’s knowledge, attitudes, and practices toward painless or epidural labor without incorporating spousal perspectives or couple-level dynamics [18,19]. Moreover, these KAP studies were conducted in settings that differ from the sociocultural and health-system context of central China, which limits the direct applicability of their findings to Wuhan. First, existing research predominantly focuses on pregnant women while overlooking spousal influence in decision-making processes. Second, studies frequently generalize findings across heterogeneous regions without considering local sociocultural contexts and healthcare system variations. Third, a paucity of data exists from central China, particularly Wuhan, a major medical hub exhibiting significant intra-urban disparities in maternal healthcare resources. In contrast to previous KAP studies, our study simultaneously includes both pregnant women and their husbands and focuses specifically on Wuhan, thereby addressing the lack of spouse-related evidence and the scarcity of region-specific data from central China.

This study addressed these gaps by examining KAP toward labor analgesia among both pregnant women and their husbands in Wuhan. We hypothesized that: (1) Greater knowledge is correlated with more positive attitudes towards labor analgesia; (2) Higher knowledge levels are associated with increased engagement in practices; and (3) Positive attitudes are positively associated with practices of labor analgesia. The findings can provide actionable insights for healthcare providers and policymakers to better support the adoption of pain relief during childbirth.

## Material and methods

### Study design and participants

This cross-sectional study was conducted in Wuhan between October 18, 2024, and April 3, 2025. Participants included pregnant women and their spouses residing in the city. The study enrolled pregnant women (1) with uncomplicated singleton pregnancies planning vaginal delivery, (2) with both partners required to be at least 18 years of age, and (3) capable of providing informed consent. Participants were excluded (1) if the pregnancy involved severe complications or planned cesarean delivery, (2) if either partner lacked decision-making capacity, or (3) if cognitive or psychiatric impairments precluded meaningful participation. The spouses of recruited pregnant women were further invited to the survey.

The study was approved by the Medical Ethics Committee of Wuhan Children’s Hospital (2024R116-E01; approval date: October 10, 2024). All participants were informed about the study protocol and provided electronic informed consent on the first page of the questionnaire, where they were required to select agreement before proceeding. All procedures were performed in accordance with the ethical standards laid down in the 1964 Declaration of Helsinki and its later amendments.

### Questionnaire design

The questionnaire was designed based on the *Chinese Expert Consensus on Neuraxial Labor Analgesia (2020 Edition)* [20]. Subsequently, it was revised under the guidance of two anesthesiologists and two obstetricians, all chief physicians from Wuhan Children’s Hospital, affiliated with Huazhong University of Science and Technology. They provided comprehensive support throughout the development and distribution process. The pilot study yielded 31 completed questionnaires from pregnant women, with 30 retained as valid responses. All 30 spouse questionnaires met the inclusion criteria when applying parallel quality thresholds. Reliability analysis demonstrated strong internal consistency for the women’s questionnaire, with Cronbach’s α of 0.863. The spouses’ section also showed acceptable reliability, with Cronbach’s α of 0.781.

To further evaluate the construct validity and sampling adequacy of the instrument, we performed analysis using the formal survey data from 549 pregnant women. A confirmatory factor analysis (CFA) was performed to validate the questionnaire’s structure by testing if the measured items fit the theoretical constructs (Figure S1). The CFA model demonstrates good fit to the data, with acceptable chi-square minimum/degrees of freedom (CMIN/DF), good root mean square error of approximation (RMSEA), and strong incremental fit indices (incremental fit index (IFI), Tucker-Lewis index (TLI), and comparative fit index (CFI) all around 0.91) (Table S1). Thus, the model is statistically well-supported and provides credible evidence that the hypothesized KAP factor structure reasonably represents the observed data. In the CFA, all KAP items loaded significantly on their respective latent factors, indicating that most items were good to strong indicators of their underlying constructs (Table S2). Additionally, the Kaiser-Meyer-Olkin (KMO) value based on the 549 cases was 0.937 (*p* < 0.001), falling in the excellent range (>0.90), meaning there is a high degree of common variance among items and the correlation matrix is very suitable for factor analysis. The associated test of sampling adequacy being statistically significant (*p* < 0.001) supports the conclusion that the observed correlations are unlikely to be random and that factor analysis is appropriate for the dataset. The pilot participants were not included in the formal analysis.

The final questionnaire for pregnant women, written in Chinese, included four sections: demographic information and separate dimensions for knowledge, attitudes, and practices. The knowledge section contained 10 items, with items K1-K9 ‘Very familiar’ scored for 2 points, ‘Heard of it’ for 1 point, and ‘Unfamiliar’ for 0 points. Item K10 was an open-ended item asking where participants had learned about labor analgesia; it was not scored. The score for this section ranged from 0 to 18. The attitude section consisted of 11 items evaluated on a 5-point Likert scale ranging from ‘Strongly disagree’ (1 point) to ‘Strongly agree’ (5 points). Item A3 was reversely scored. The score for this section ranged from 11 to 55. The practice section included 5 items, also rated on a 5-point Likert scale from ‘Never’ (1 point) to ‘Always’ (5 points). The score for this section ranged from 5 to 25.

The husband questionnaire included two sections: demographic characteristics and assessment of knowledge, willingness, and involvement in labor analgesia decision-making. It comprised eight total questions, with items 1–6 utilizing a 5-point Likert scale (total score range: 6–30). The final questionnaire was translated into an English version (see the appendix named ‘Questionnaire’) [21,22].

KAP scores were calculated as the percentage of correct answers (0-100%). Based on modified Bloom-type criteria commonly used in KAP research, scores >70% were classified as ‘good’ and scores <70% as ‘poor’.

### Data collection and procedures

The questionnaire was distributed using both online and offline methods. Before accessing the questionnaire, participants were required to tick an electronic consent option on the first page (and similarly on paper forms), and only those who selected agreement were allowed to proceed. An online version was created through Wenjuanxing (Questionnaire Star), a WeChat-based survey platform. A QR code linking to the questionnaire was shared in WeChat groups maintained by obstetric departments for pregnant women. Participants accessed and completed the questionnaire by scanning the code through WeChat. Administered separately from the wife questionnaire. In the women’s questionnaire, participants entered their own phone number, while husbands entered their wives’ phone number; these entries were used to match the two questionnaires during analysis. To maintain data integrity, each IP address was allowed only one submission for each questionnaire (women and spouses), and all questions were mandatory. For those attending outpatient prenatal visits, trained researchers conducted face-to-face surveys and distributed paper-based questionnaires. All completed questionnaires were reviewed by the research team for completeness, internal consistency, and logical accuracy to ensure data quality. Those with incomplete consent, incomplete responses, data entry errors, age ineligibility, or incorrect trap question responses were excluded.

**Figure 1.**
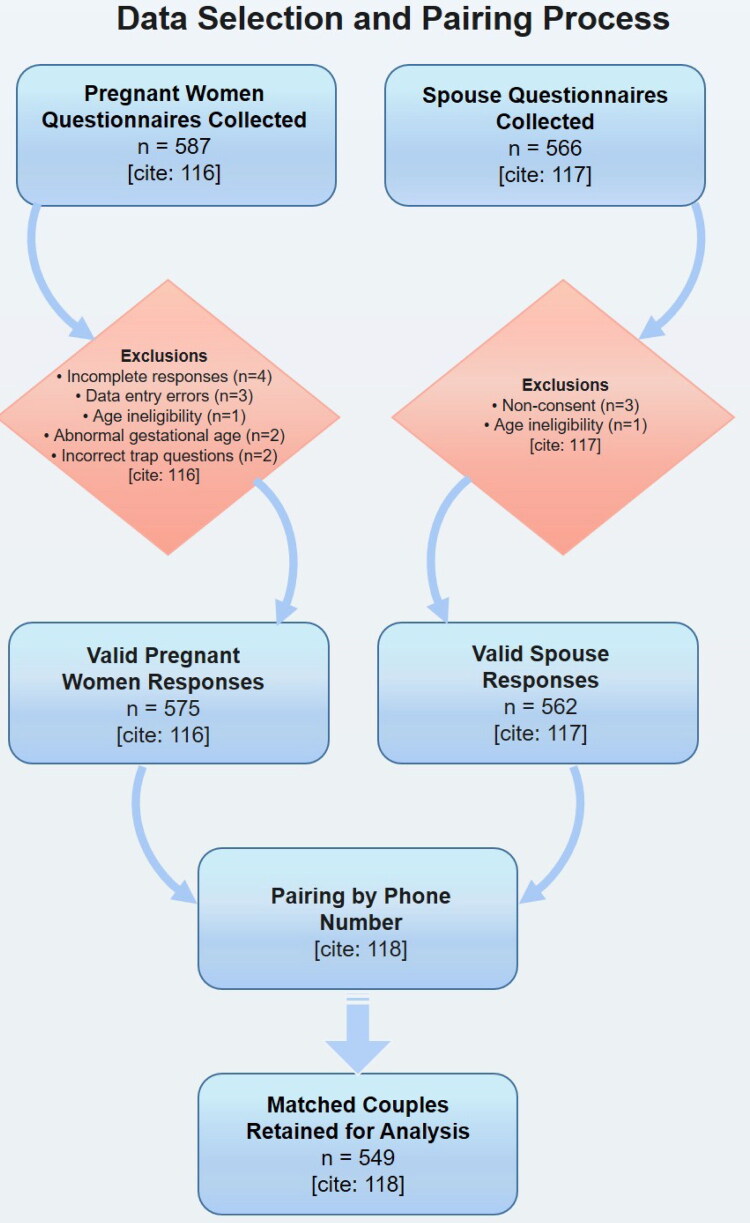
Study flowchart.

The study collected 587 questionnaires from pregnant women, with 12 excluded due to incomplete responses (*n* = 4 completed in under 90 s), data entry errors (*n* = 3 with incorrect field entries), age ineligibility (*n* = 1 under 18 years), abnormal gestational age (*n* = 2), and incorrect trap question responses (*n* = 2), yielding 575 valid responses. Among spouses, 566 questionnaires were obtained, with exclusions for non-consent (*n* = 3) and age ineligibility (*n* = 1), resulting in 562 valid responses (Figure 1). After pairing participants by phone number, 549 matched couples were retained for analysis.

### Sample size calculation

The sample size calculation followed a commonly used formula in KAP studies, which is suitable for cross-sectional surveys [23–25]:

$$n={\left(\frac{Z_{1-\frac{\alpha }{2}}}{\delta }\right)}^{2}\times p\times (1-p)$$

where n denotes the sample size, and p was assumed to be 0.5 to ensure the maximum sample size. α, also known as the type I error, was set to 0.05. In this case, $Z_{1-\frac{\alpha }{2}}=1.96$, and δ represents the effect sizes between groups, which was determined as 0.05. Assuming an effective questionnaire recovery rate of 80%, the final target is to collect at least 480 completed questionnaires from pregnant women. The spouses of the enrolled pregnant women were invited to participate, but their participation was not mandatory for the participation of the pregnant women.

### Statistical methods

Statistical analyses were conducted using SPSS version 26.0 and AMOS version 24.0 (IBM, Armonk, NY, USA). Score distributions were tested for normality. KAP data were expressed as mean ± standard deviation (SD). Categorical variables were summarized as frequencies and percentages. For continuous variables, the Student’s t-test was used for normally distributed data, and the Mann-Whitney U test for non-normal data. Comparisons involving three or more groups employed ANOVA for normally distributed variables with homogeneity of variances and the Kruskal-Wallis test for non-normally distributed data. Spearman correlation was adopted to assess the relationships among knowledge, attitude, practice, and spouse scores.

To identify factors associated with practice scores among pregnant women, univariate and multivariate logistic regression analyses were performed. Practice scores were dichotomized using *a* ≥ 70% cutoff of the total score according to published research [24]. Variables with *p* < 0.05 in univariate analyses were included in the multivariate model.

Structural equation modeling (SEM), based on the KAP theoretical framework, was used to examine the direct and indirect associations among knowledge, attitude, practice, and spouse scores. Model fit was evaluated using the following indices: CMIN/DF, RMSEA, IFI, TLI, and CFI. While bivariate correlations and multiple regression were used to describe simple associations between observed variables, they cannot account for measurement error in the multi-item scales nor test the hypothesized network of direct and indirect pathways simultaneously. Structural equation modeling was therefore employed to (i) specify latent constructs from multiple indicators, (ii) estimate direct and indirect effects among constructs in a single model, and (iii) evaluate the overall fit of the proposed theoretical model to the data. This approach provides a more rigorous, theory-driven assessment of the relationships than would be possible with separate correlation and regression analyses alone.

A two-tailed P value < 0.05 was considered statistically significant.

## Results

### Demographic characteristics

The questionnaire demonstrated excellent internal consistency (Cronbach’s α = 0.930 for the whole study sample, excluding the pilot participants). Among the pregnant women surveyed, 85.61% were under 35 years of age, 75.05% resided in urban areas, and 45.72% held a bachelor’s degree. A plurality reported a monthly household income of 5000-10,000 CNY (equivalent to 690–1380 USD) (43.53%). Most were in the third trimester (28 weeks to delivery; 79.78%) and nulliparous (74.13%). Notably, 72.86% intended to choose labor analgesia for their current delivery. Their husbands had a mean age of 31.89 ± 3.90 years. In addition, 82.89% of husbands were under 35 years of age. Of them, 47.72% held a bachelor’s degree, and 27.32% were employed as general clerical staff or in related occupations. The average score on the spouse questionnaire was 21.69 ± 3.78 (Table 1).

**Table 1.** Baseline characteristics and KAP scores of pregnant women (*n* = 549) and their husbands (*n* = 549).

Variables	n (%)	Knowledge		Attitude		Practice	
		Mean ± SD	*P*	Mean ± SD	*P*	Mean ± SD	*P*
**Pregnant women’s information**		7.19 ± 4.32		45.66 ± 5.91		11.94 ± 3.96	
**Age (Year)**	30.54 ± 3.78		0.463		0.229		0.914
<35	470 (85.61)	7.24 ± 4.32		45.71 ± 6.1		11.95 ± 4.01	
≥35 (advanced maternal age)	79 (14.39)	6.94 ± 4.3		45.34 ± 4.6		11.84 ± 3.61	
**Place of residence**			<0.001		<0.001		<0.001
Rural or suburban	137 (24.95)	5.2 ± 3.87		42.77 ± 6.85		10.94 ± 3.43	
Urban	412 (75.05)	7.86 ± 4.26		46.61 ± 5.23		12.27 ± 4.07	
**Education level**			<0.001		<0.001		<0.001
Associate degree or below	229 (41.71)	5.62 ± 3.92		43.69 ± 6.51		10.66 ± 3.47	
Bachelor’s degree	251 (45.72)	7.98 ± 4.17		46.65 ± 4.86		12.43 ± 3.95	
Master’s degree or above	69 (12.57)	9.54 ± 4.27		48.58 ± 5.18		14.36 ± 4.02	
**Current occupation**			<0.001		0.003		<0.001
Professionals	111 (20.22)	8.81 ± 4.59		47.33 ± 5.31		13.5 ± 4.06	
General clerical staff and related personnel	180 (32.79)	7.39 ± 4.17		46.08 ± 5.05		12.01 ± 3.78	
Commercial or service industry workers	75 (13.66)	5.67 ± 3.83		44.91 ± 5.39		12.08 ± 3.75	
Other	183 (33.33)	6.64 ± 4.17		44.53 ± 6.91		10.85 ± 3.83	
**Monthly income per capita (Chinese Yuan)**			<0.001		<0.001		<0.001
<5000	137 (24.95)	5.18 ± 3.39		42.31 ± 6.92		10.82 ± 3.35	
5000-10,000	239 (43.53)	6.75 ± 3.88		45.75 ± 4.94		11.42 ± 3.53	
10,000–20,000	129 (23.5)	9.23 ± 4.56		47.78 ± 4.95		13.58 ± 4.52	
>20,000	44 (8.01)	9.86 ± 4.71		49.34 ± 4.74		13.36 ± 4.3	
**Current gestational age**			0.731		0.249		0.703
1–12 weeks	28 (5.1)	7.61 ± 3.59		43.89 ± 6.16		12.39 ± 3.94	
13–27 weeks	83 (15.12)	7.31 ± 5.02		45.63 ± 6.35		12.01 ± 4	
≥28 weeks	438 (79.78)	7.14 ± 4.22		45.77 ± 5.8		11.89 ± 3.96	
**Previous pregnancy(ies)**			0.539		0.858		0.019
Once	407 (74.13)	7.12 ± 4.31		45.62 ± 6.11		11.75 ± 3.97	
Twice or more	142 (25.87)	7.39 ± 4.33		45.77 ± 5.31		12.46 ± 3.89	
**Labor analgesia during previous delivery (For women who have previously given birth only)**			0.01		0.006		<0.001
Yes	65 (11.84)	8.45 ± 4.26		47.18 ± 4.78		13.91 ± 3.53	
No	77 (14.03)	6.51 ± 4.2		44.57 ± 5.47		11.23 ± 3.77	
**Rate the previous labor analgesia experience**			0.439		0.008		0.009
Satisfied	57 (87.69)	8.56 ± 4.21		47.79 ± 4.45		14.23 ± 3.02	
Dissatisfied	8 (12.31)	7.63 ± 4.87		42.88 ± 5.11		11.63 ± 5.83	
**Labor analgesia for this delivery**			<0.001		<0.001		<0.001
Yes	400 (72.86)	7.91 ± 4.35		47.05 ± 5.15		12.58 ± 3.94	
No	4 (0.73)	7.75 ± 6.4		44 ± 6.68		10.75 ± 4.43	
Depends on the situation	145 (26.41)	5.21 ± 3.51		41.86 ± 6.2		10.19 ± 3.45	
**Type of medical insurance**			<0.001		<0.001		<0.001
Only social medical insurance	358 (65.21)	6.94 ± 4.04		45.69 ± 5.19		11.53 ± 3.65	
Only commercial medical insurance	2 (0.36)	8 ± 2.83		43.5 ± 0.71		12 ± 0	
Both social and commercial medical insurance	156 (28.42)	8.6 ± 4.61		47.64 ± 5.22		13.38 ± 4.43	
No insurance	33 (6.01)	3.27 ± 2.74		36 ± 7.05		9.48 ± 2.41	
**Spouse information**							
**Age**	31.89 ± 3.90						
**Education level**			<0.001		<0.001		<0.001
Associate degree or below	193 (35.15)	5.93 ± 4.02		43.45 ± 6.37		10.51 ± 3.47	
Bachelor’s degree	262 (47.72)	7.28 ± 4.15		46.26 ± 5.24		12.23 ± 3.75	
Master’s degree or above	94 (17.12)	9.54 ± 4.38		48.5 ± 5.05		14.03 ± 4.37	
**Current occupation**			<0.001		<0.001		<0.001
Leaders of government agencies, Party organizations, or enterprises/institutions	53 (9.65)	8.51 ± 3.98		47.6 ± 5.21		12.89 ± 4.73	
Professionals	130 (23.68)	8.34 ± 4.45		47.4 ± 5.42		13.24 ± 4.14	
General clerical staff and related personnel	150 (27.32)	7.19 ± 4.25		46.19 ± 5.18		11.91 ± 3.86	
Commercial or service industry workers	83 (15.12)	6.33 ± 4.15		43.31 ± 6.69		11.45 ± 3.86	
Operators of production and transportation equipment and related personnel	44 (8.01)	5.2 ± 3.73		41.3 ± 6.9		10.14 ± 3.02	
Other	89 (16.21)	6.54 ± 4.26		45.39 ± 5.1		10.87 ± 3.06	
**Spouse questionnaire score**	21.69 ± 3.78						

Professionals included professions such as teachers, doctors, engineers, and writers. Gestational age could be categorized as early pregnancy (1–12 weeks), middle pregnancy (13–27 weeks), and late pregnancy (28 weeks to delivery).

### Knowledge, attitude, and practice dimensions

The mean knowledge score among pregnant women was 7.19 ± 4.32. Significant differences were found in knowledge scores across factors, such as residence (*p* < 0.001), education level (*p* < 0.001), intention to choose labor analgesia (*p* < 0.001), spousal education level (*p* < 0.001), and current occupation (*p* < 0.001) (Table 1). Regarding sources of information, 44.99% cited the internet or social media, followed by healthcare professionals (32.6%) and family or friends (21.13%) (Table S3).

The mean attitude score was 45.66 ± 5.91. Attitude scores varied significantly by factors, including residence (*p* < 0.001), current occupation (*p* = 0.003), intention to choose labor analgesia (*p* < 0.001), spousal education level (*p* < 0.001), and spousal occupation (*p* < 0.001) (Table 1). Positive response rates across attitude items ranged from 48.63% to 96.54% (Table S4).

The mean practice score was 11.94 ± 3.96. Practice scores showed significant variation by factors, such as education level (*p* < 0.001), monthly income (*p* < 0.001), choice of labor analgesia (*p* < 0.001), spousal education (*p* < 0.001), and spousal occupation (*p* < 0.001) (Table 1). Reported engagement in relevant practices ranged from 7.47% to 24.22% (Table S5).

Among participating husbands, only 28.05% demonstrated an understanding of the concept and related aspects of labor analgesia. When asked about their support for their wives’ preferences, 82.87% expressed willingness to endorse their wives’ decision to opt for labor analgesia during labor. However, only 34.98% reported no concern regarding potential adverse effects on neonatal health. In terms of perceived benefits, 83.97% believed that labor analgesia could relieve labor pain, followed by 74.13% who noted its ability to reduce fear of childbirth, and 69.76% who believed it increased comfort during delivery. Despite these perceived advantages, concerns remained prevalent: 73.41% of husbands reported unfamiliarity with the technology involved, and 59.56% expressed worry that it might affect the health of both mother and child (Table 2).

**Table 2.** Knowledge, willingness, and decision-making of husbands of pregnant women in Wuhan regarding labor analgesia.

	N (%)				
	Very well	Well	Neutral	Not well	Not at all
1. How well do you understand the concept and related information about labor analgesia? (P)	10 (1.82)	144 (26.23)	204 (37.16)	145 (26.41)	46 (8.38)
	**Strongly agree**	**Agree**	**Neutral**	**Disagree**	**Strongly disagree**
2. Do you think labor analgesia is safe? (P)	49 (8.93)	270 (49.18)	161 (29.33)	57 (10.38)	12 (2.19)
3. Do you believe labor analgesia can help reduce your wife’s pain during delivery? (P)	91 (16.58)	305 (55.56)	116 (21.13)	33 (6.01)	4 (0.73)
4. Are you concerned that labor analgesia may have negative effects on the baby’s health? (N)	30 (5.46)	143 (26.05)	184 (33.52)	170 (30.97)	22 (4.01)
5. If your wife wishes to choose labor analgesia during labor, would you support her decision? (P)	207 (37.7)	248 (45.17)	82 (14.94)	12 (2.19)	
6. Are you willing to pay extra for your wife to receive labor analgesia services? (P)	171 (31.15)	273 (49.73)	94 (17.12)	11 (2)	
	**Relieves labor pain**	**Reduces fear of childbirth**	**Increases comfort during delivery**	**Shortens the duration of labor**	**Other**
7. What benefits do you think labor analgesia offers to pregnant women?	461 (83.97)	407 (74.13)	383 (69.76)	188 (34.24)	5 (0.91)
	**May affect the health of the mother and baby**	**Not familiar with the technology of labor analgesia**	**Concerned about the hospital’s equipment and technical capabilities**	**Concerned about increased delivery costs**	**Other**
8. What concerns do you have about the potential side effects or risks of labor analgesia?	327 (59.56)	403 (73.41)	310 (56.47)	61 (11.11)	3 (0.55)

### Spearman correlation analysis

Spearman correlation analysis revealed significant positive correlations between knowledge and attitude (*r* = 0.543, *p* < 0.001), knowledge and practice (*r* = 0.528, *p* < 0.001), and attitude and practice (*r* = 0.386, *p* < 0.001). Spouse scores were also positively correlated with knowledge (*r* = 0.333, *p* < 0.001), attitude (*r* = 0.433, *p* < 0.001), and practice (*r* = 0.373, *p* < 0.001) (Table S6).

### Multivariate logistic regression analysis

Multivariate logistic regression identified several factors significantly associated with higher practice scores among pregnant women. These included knowledge score (OR = 1.347, 95% CI: 1.213–1.497, *p* < 0.001), husbands being employed as a leader in government, party organizations, or enterprises/institutions (OR = 6.211, 95% CI: 1.098–35.125, *p* = 0.039), and husbands working in the commercial or service industry (OR = 8.526, 95% CI: 1.264–57.523, *p* = 0.028) **(**Table 3).

**Table 3.** Univariate and multivariate logistic regression of pregnant women in Wuhan regarding labor analgesia.

Practice (<70% the total score as reference)	Univariate logistic regression		Multivariate logistic regression	
	OR (95%CI)	P	OR (95%CI)	P
**Knowledge score**	1.410 (1.299–1.530)	<0.001	1.347 (1.213–1.497)	<0.001
**Attitude score**	1.240 (1.153–1.333)	<0.001	1.041 (0.949–1.142)	0.398
**Spouse score**	1.168 (1.068–1.278)	<0.001	1.029 (0.908–1.167)	0.651
**Age (Year)**				
<35	ref			
≥35 (advanced maternal age)	0.654 (0.251–1.704)	0.385		
**Place of residence**				
Rural or suburban	ref		ref	
Urban	2.544 (1.058–6.116)	0.037	0.709 (0.231–2.175)	0.547
**Education level**				
Associate degree or below	ref		ref	
Bachelor’s degree	4.295 (1.734–10.636)	0.002	1.728 (0.520–5.747)	0.372
Master’s degree or above	12.151 (4.567–32.324)	<0.001	2.548 (0.583–11.127)	0.214
**Current occupation**				
Professionals	4.276 (1.941–9.423)	<0.001	1.992 (0.711–5.579)	0.190
General clerical staff and related personnel	1.236 (0.520–2.937)	0.632	1.208 (0.420–3.474)	0.726
Commercial or service industry workers	1.236 (0.408–3.745)	0.708	1.671 (0.386–7.232)	0.492
Other	ref		ref	
**Monthly income per capita (Chinese Yuan)**				
<5000	ref		ref	
5000-10,000	2.155 (0.591–7.862)	0.245	0.986 (0.239–4.057)	0.984
10,000–20,000	11.275 (3.321–38.282)	<0.001	1.891 (0.453–7.883)	0.382
>20,000	11.486 (2.952–44.687)	<0.001	1.142 (0.209–6.221)	0.878
**Current gestational age**				
1–12 weeks	ref			
13–27 weeks	1.273 (0.328–4.937)	0.727		
≥28 weeks	0.724 (0.208–2.517)	0.611		
**Previous pregnancy(ies)**				
Once	ref			
Twice or more	0.621 (0.293–1.314)	0.213		
**Spouse age**	0.994 (0.922–1.073)	0.883		
**Spouse’s education level**				
Associate degree or below	ref		ref	
Bachelor’s degree	2.857 (1.135–7.188)	0.026	1.575 (0.446–5.561)	0.481
Master’s degree or above	8.966 (3.479–23.108)	<0.001	1.865 (0.389–8.938)	0.436
**Current occupation**				
Leaders of government agencies, Party organizations, or enterprises/institutions	10.116 (2.123–48.215)	0.004	6.211 (1.098–35.125)	0.039
Professionals	8.381 (1.913–36.725)	0.005	3.691 (0.683–19.960)	0.129
General clerical staff and related personnel	2.451 (0.509–11.807)	0.264	2.431 (0.437–13.527)	0.310
Commercial or service industry workers	4.007 (0.808–19.871)	0.089	8.526 (1.264–57.523)	0.028
Operators of production and transportation equipment and related personnel	1.012 (0.089–11.469)	0.993	4.960 (0.334–73.585)	0.245
Other	ref		ref	

Professionals included professions such as teachers, doctors, engineers, and writers. Gestational age could be categorized as early pregnancy (1–12 weeks), middle pregnancy (13–27 weeks), and late pregnancy (28 weeks to delivery).

### SEM and mediation analysis

**Figure 2.**
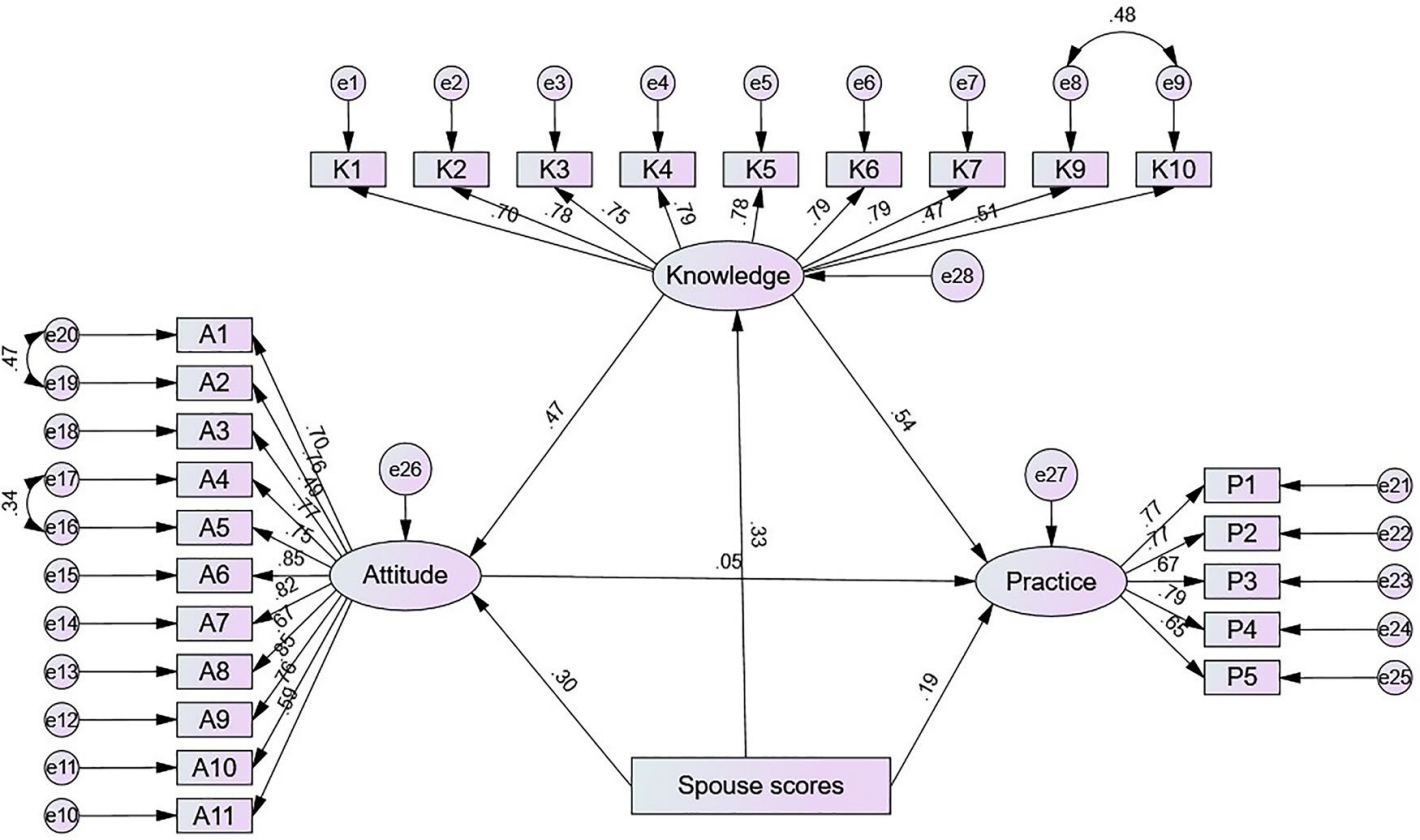
SEM results of KAP scores. All variables are observed variables. Direction of causality is indicated by single-headed arrows, and interactions between variables are denoted by two-headed arrows. The standardized path coefficients are presented alongside the arrows.

SEM demonstrated good model fit (CMIN/DF = 3.999, RMSEA = 0.074, IFI = 0.900, TLI = 0.888, CFI = 0.900) (Table S7). SEM confirmed significant direct paths from knowledge to attitude (standardized coefficient = 0.473, *p* < 0.001) and from knowledge to practice (standardized coefficient = 0.544, *p* < 0.001). Additionally, spouse scores were significantly associated with knowledge (standardized coefficient = 0.326, *p* < 0.001), attitude (standardized coefficient = 0.299, *p* < 0.001), and practice (standardized coefficient = 0.186, *p* < 0.001) (Table S8 and Figure 2). Mediation analysis further supported these relationships. Knowledge exerted direct effects on both attitude (β = 0.473, 95% CI: 0.388–0.542, *p* = 0.014) and practice (β = 0.544, 95% CI: 0.439–0.650, *p* = 0.009). Spouse scores also had direct effects on knowledge (β = 0.326, 95% CI: 0.231–0.403, *p* = 0.008), attitude (β = 0.299, 95% CI: 0.159–0.362, *p* = 0.041), and practice (β = 0.186, 95% CI: 0.107–0.281, *p* = 0.009). Moreover, indirect effects of spouse scores were observed on both attitude (β = 0.154, 95% CI: 0.110–0.204, *p* = 0.005) and practice (β = 0.198, 95% CI: 0.130–0.262, *p* = 0.007) (Table 4).

**Table 4.** Results of direct and indirect effects analysis in the SEM model.

Model paths	Standardized total effects		Standardized direct effects		Standardized indirect effects	
	β (95%CI)	P	β (95%CI)	P	β (95%CI)	P
Knowledge → Attitude	0.473 (0.388–0.542)	0.014	0.473 (0.388–0.542)	0.014		
Knowledge → Practice	0.566 (0.488–0.658)	0.006	0.544 (0.439–0.650)	0.009	0.022 (−0.030-0.067)	0.404
Attitude → Practice	0.046 (−0.067-0.141)	0.419	0.046 (−0.067-0.141)	0.419		
Spouse scores → Knowledge	0.326 (0.231–0.403)	0.008	0.326 (0.231–0.403)	0.008		
Spouse scores → Attitude	0.453 (0.360–0.514)	0.044	0.299 (0.159–0.362)	0.041	0.154 (0.110–0.204)	0.005
Spouse scores → Practice	0.383 (0.300–0.458)	0.016	0.186 (0.107–0.281)	0.009	0.198 (0.130–0.262)	0.007

## Discussion

This cross-sectional survey assessed the knowledge, attitudes, and practices (KAP) of pregnant women and their husbands in Wuhan concerning labor analgesia. Findings indicated that pregnant women generally demonstrated limited knowledge and practice but maintained positive attitudes toward labor analgesia, whereas their husbands exhibited adequate knowledge, willingness, and involvement. Among pregnant women, higher knowledge levels, spousal leadership positions, and employment in commercial or service sectors were positively associated with better practices. Mediation analysis identified significant direct effects of knowledge on both attitude and practice, as well as direct effects of husbands’ scores on KAP. Additionally, husbands’ scores exerted indirect effects on women’s attitudes and practices.

In the knowledge section, although a proportion of pregnant women in our study demonstrated some understanding of the basic concept of labor analgesia, their overall knowledge remained limited and fragmented. Similarly, an Ethiopian study reported that 6only 8% of pregnant women were aware of labor analgesia, and among these, just 60.6% had heard about it during their current pregnancy, with very few having prior experience or comprehensive understanding of the method [25]. Few participants were able to recognize the labor analgesia contraindications in the present study, such as severe lumbar spine disorders or prior lumbar surgery. One potential explanation is the obstetric-focused nature of routine prenatal consultations, which prioritize obstetric assessments over in-depth analgesia counseling. Some information may also be left unassimilated if the delivery process progresses quickly and decisions must be made quickly. Additionally, fewer than half of respondents accurately identified potential side effects. This knowledge gap indicated an incomplete perception of procedural safety, consistent with a Saudi Arabian study in which 42.6% of pregnant women questioned whether epidurals could cause paraplegia [26]. To improve understanding, future efforts should deliver comprehensive, balanced information *via* multimedia tools and community-based prenatal education.

In the attitude dimension, most respondents held a positive attitude towards government subsidies for labor analgesia to improve accessibility. Chinese women supported policy-based expansion of labor analgesia services and believed that insurance reimbursement would encourage uptake [27]. This finding reflected a strong public demand for reducing economic barriers to labor analgesia and the widespread recognition of the procedure’s benefits. However, less than half correctly rejected the misconception that labor analgesia adversely affects infant health. In other words, over half of pregnant women either held this misconception or were uncertain of the safety. Scientific evidence has confirmed that epidural analgesia poses no significant risks to neonatal or childhood development outcomes [28]. Health professionals should emphasize evidence-based reassurance about fetal safety while correcting misunderstandings related to ‘unnatural birth’ narratives.

In the practice dimension, the most commonly reported behavior was independently seeking information on labor analgesia, though overall engagement remained limited. This reflected the digital health-seeking behaviors among Chinese populations, where widespread smartphone use facilitates access to health-related content [29]. Conversely, only 7.47% attended health education sessions. In China, antenatal care is typically delivered in high-volume clinical settings, leaving minimal time for structured education. Also, childbirth education is often undervalued in institutional settings, reducing opportunities for comprehensive learning [30]. Online engagement was similarly low, with 8.56% sharing experiences about labor analgesia on social media. This limited participation likely stemmed from privacy concerns. Social norms may discourage disclosure of personal experiences, especially in contexts where fear of judgment prevails. Comparable patterns have been observed in other populations; for instance, a study of Western Australian women found that many were hesitant to share positive birth stories due to concerns about making others feel inadequate [31]. To enhance practical engagement, peer communication in online forums-ensuring anonymity and data security-may provide a psychologically safe environment for information exchange.

This study reveals significant knowledge deficits and concerns among expectant fathers regarding labor analgesia. Nearly a quarter demonstrated basic understandings of the procedure. Besides, most husbands reported limited familiarity with the technique and expressed concerns about potential harm to both mother and infant. These findings corroborated previous research on paternal perceptions of labor analgesia. An Israeli study found that while 64.1% of expectant fathers initially supported epidural use, their acceptance rates remained substantially lower than those of their maternal counterparts [32]. The knowledge gap likely derives from constrained participation in prenatal education and reliance on informal information sources [33]. Research shows that paternal involvement during labor, including support for epidurals, can reduce maternal stress and improve childbirth experiences [34]. To address these challenges, tailored interventions targeting husbands are essential. Antenatal programs should actively include male partners, providing clear information on analgesic techniques and their safety profile.

Our results demonstrated positive correlations among KAP, aligning with the theoretical KAP framework. SEM further confirmed the direct effects of knowledge on both attitude and practice. These consistent findings underscore the critical role of knowledge in shaping attitudes and behaviors of labor analgesia. Similar patterns have been reported in prior studies. For instance, research on Chinese pregnant women showed that knowledge strongly influenced beliefs and behaviors related to perinatal self-protection [35]. Another study among Chinese midwives identified moderately positive correlations between KAP in managing birthing positions [36]. However, a non‑significant path was noted from attitude to practice within SEM analysis. Several reasons may impede the attitude-practice transitions, including the affordability of neuraxial analgesia and the timely access to intrapartum care [37]. Notably, spouse scores also exerted significant indirect effects on attitude and practice, indicating that husband engagement enhances maternal outcomes beyond mere knowledge transmission. Couple-focused counseling and male-inclusive antenatal classes are recommended to foster male involvement and spousal support. In the logistic regression, women employed in leadership within government, enterprises, or the commercial and service sectors were more likely to engage in labor analgesia. These occupational influences likely reflect greater access to health information and increased decision-making autonomy.

This study had several limitations. First, the findings may not be generalizable beyond Wuhan. Second, self-reported data may introduce social desirability bias, potentially affecting the accuracy [38]. Third, the cross-sectional design precluded causal inference. Fourth, the extremely wide confidence intervals for the subgroups of government/organizational leaders and husbands working in commerce or services indicate limited precision of these estimates. Nonetheless, this study was the first to examine the KAP of both pregnant women and their husbands regarding labor analgesia in Wuhan. These findings elucidate critical deficiencies in knowledge and practice surrounding labor analgesia and emphasize the necessity of tailored educational strategies that address both pregnant women and their spouses to promote evidence‑based choices during labor. Future studies should explore these KAP gaps in diverse settings and use longitudinal or interventional designs to clarify causal pathways and to test strategies for improving informed uptake of labor analgesia.

## Conclusion

Pregnant women in Wuhan exhibited poor knowledge and practices despite positive attitudes towards labor analgesia. In addition, their spouses showed good knowledge/willingness/involvement towards labor analgesia. Spouse scores showed indirect effects on both attitudes and practices, reinforcing the importance of partner engagement. These findings highlight the need for educational and behavioral strategies-emphasizing contraindications, procedural safety, health education, and online platforms-to improve KAP among both pregnant women and their husbands.

## Supplementary Material

Supplementary figures.docx

Supplementary tables.docx

STROBE Checklist.doc

## Data Availability

The authors confirm that the data supporting the findings of this study are available within the article and its supplementary materials.
