# Supplementary figures and images for: Labor analgesia: knowledge, attitudes, and practices among pregnant women and husbands in Wuhan: a cross-sectional study
Source: Ann Med. 2026 May 12;58(1):2670069. doi: 10.1080/07853890.2026.2670069 (PMC13173574; doi:10.1080/07853890.2026.2670069)

**Figure S1. Confirmatory factor analysis.**


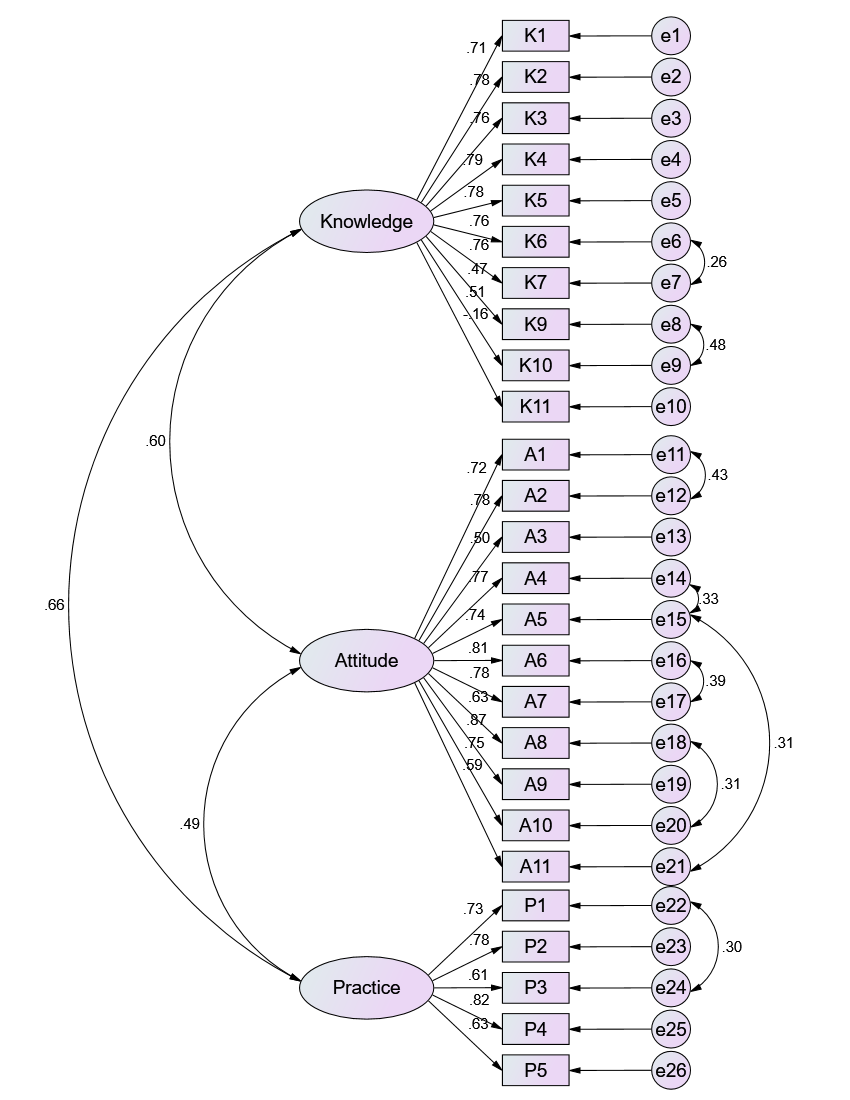

Supplement: Supplementary figures.docx [file IANN_A_2670069_SM0363.docx]
