# Supplementary material for: Labor analgesia: knowledge, attitudes, and practices among pregnant women and husbands in Wuhan: a cross-sectional study
Source: Ann Med. 2026 May 12;58(1):2670069. doi: 10.1080/07853890.2026.2670069 (PMC13173574; doi:10.1080/07853890.2026.2670069)
Supplement: Supplementary tables.docx [file IANN_A_2670069_SM0362.docx]

**Table S1. Fit indices of the confirmatory factor analysis**

| Indicators | Reference | Actual |
| --- | --- | --- |
| CMIN/DF | 1-3: Excellent, 3-5: Good | 3.516 |
| RMSEA | <0.08: Good | 0.068 |
| IFI | >0.8: Good | 0.917 |
| TLI | >0.8: Good | 0.905 |
| CFI | >0.8: Good | 0.916 |

Abbreviations: CMIN/DF, chi-square minimum/degrees of freedom; RMSEA, root mean square error of approximation; IFI, incremental fit index; TLI, Tucker-Lewis index; CFI, comparative fit index

**Table S2. Confirmatory factor analysis**

|  |  |  | **Estimate** | **Standardized Estimate** | **S.E.** | **C.R.** | **P** |
| --- | --- | --- | --- | --- | --- | --- | --- |
| K1 | <--- | Knowledge | 1.000 | 0.709 |  |  |  |
| K2 | <--- | Knowledge | 1.586 | 0.782 | 0.091 | 17.353 | <0.001 |
| K3 | <--- | Knowledge | 1.421 | 0.756 | 0.085 | 16.778 | <0.001 |
| K4 | <--- | Knowledge | 1.554 | 0.793 | 0.088 | 17.584 | <0.001 |
| K5 | <--- | Knowledge | 1.384 | 0.780 | 0.080 | 17.302 | <0.001 |
| K6 | <--- | Knowledge | 1.467 | 0.762 | 0.087 | 16.876 | <0.001 |
| K7 | <--- | Knowledge | 1.512 | 0.764 | 0.089 | 16.899 | <0.001 |
| K9 | <--- | Knowledge | 0.745 | 0.471 | 0.071 | 10.515 | <0.001 |
| K10 | <--- | Knowledge | 0.911 | 0.513 | 0.080 | 11.447 | <0.001 |
| K11 | <--- | Knowledge | -0.406 | -0.157 | 0.115 | -3.517 | <0.001 |
| A1 | <--- | Attitude | 1.000 | 0.723 |  |  |  |
| A2 | <--- | Attitude | 1.004 | 0.779 | 0.043 | 23.565 | <0.001 |
| A3 | <--- | Attitude | 0.913 | 0.504 | 0.080 | 11.416 | <0.001 |
| A4 | <--- | Attitude | 1.039 | 0.775 | 0.059 | 17.672 | <0.001 |
| A5 | <--- | Attitude | 0.990 | 0.737 | 0.059 | 16.763 | <0.001 |
| A6 | <--- | Attitude | 1.065 | 0.811 | 0.058 | 18.482 | <0.001 |
| A7 | <--- | Attitude | 0.984 | 0.781 | 0.055 | 17.777 | <0.001 |
| A8 | <--- | Attitude | 0.717 | 0.629 | 0.050 | 14.268 | <0.001 |
| A9 | <--- | Attitude | 1.221 | 0.868 | 0.062 | 19.817 | <0.001 |
| A10 | <--- | Attitude | 0.867 | 0.753 | 0.051 | 17.172 | <0.001 |
| A11 | <--- | Attitude | 0.944 | 0.588 | 0.071 | 13.330 | <0.001 |
| P1 | <--- | Practice | 1.000 | 0.730 |  |  |  |
| P2 | <--- | Practice | 1.041 | 0.785 | 0.062 | 16.880 | <0.001 |
| P3 | <--- | Practice | 0.766 | 0.610 | 0.048 | 15.824 | <0.001 |
| P4 | <--- | Practice | 1.059 | 0.819 | 0.061 | 17.461 | <0.001 |
| P5 | <--- | Practice | 0.858 | 0.633 | 0.062 | 13.765 | <0.001 |

**Table S3. Knowledge section of pregnant women in Wuhan regarding painless childbirth**

|  | **N (%)** | | |
| --- | --- | --- | --- |
|  | **Very familiar** | **Heard of it** | **Unfamiliar** |
| 1. Do you know what painless childbirth is? | 76 (13.84) | 414 (75.41) | 59 (10.75) |
| 1. Painless childbirth does not affect the baby's intelligence. | 123 (22.4) | 269 (49) | 157 (28.6) |
| 1. Painless childbirth can be requested voluntarily by the mother if approved by the obstetrician. | 154 (28.05) | 303 (55.19) | 92 (16.76) |
| 1. The first-line method for labor analgesia is neuraxial (epidural or spinal) analgesia. | 78 (14.21) | 255 (46.45) | 216 (39.34) |
| 1. Painless childbirth can effectively reduce the number of cesarean sections caused by unbearable pain, helping to avoid "double suffering" from failed vaginal delivery followed by cesarean section. | 103 (18.76) | 336 (61.2) | 110 (20.04) |
| 1. Painless childbirth involves an injection in the lower back and does not cause long-term back pain. | 82 (14.94) | 278 (50.64) | 189 (34.43) |
| 1. The drugs used for painless childbirth are in low concentrations and do not affect uterine contractions or physical strength during labor. | 81 (14.75) | 250 (45.54) | 218 (39.71) |
| 1. Painless childbirth may cause side effects such as nerve damage, low blood pressure, and itching. | 23 (4.19) | 140 (25.5) | 386 (70.31) |
| 1. Women with severe lumbar spine diseases or a history of lumbar surgery are not recommended to receive neuraxial labor analgesia. | 38 (6.92) | 188 (34.24) | 323 (58.83) |
| 1. Where did you learn about painless childbirth? |  |  |  |
| Doctors or medical professionals | 179 (32.6) |  |  |
| Family or friends | 116 (21.13) |  |  |
| Internet or social media | 247 (44.99) |  |  |
| Books or magazines | 5 (0.91) |  |  |
| Other | 2 (0.36) |  |  |

**Table S4. Attitude section of pregnant women in Wuhan regarding painless childbirth**

|  | **N (%)** | | | | |
| --- | --- | --- | --- | --- | --- |
|  | **Strongly agree** | **Agree** | **Neutral** | **Disagree** | **Strongly disagree** |
| 1. I believe painless childbirth is safe. (P) | 108 (19.67) | 324 (59.02) | 98 (17.85) | 18 (3.28) | 1 (0.18) |
| 1. I believe painless childbirth can help reduce pain during labor. (P) | 150 (27.32) | 326 (59.38) | 62 (11.29) | 11 (2) |  |
| 1. I am concerned that painless childbirth may negatively affect the baby's health. (N) | 13 (2.37) | 91 (16.58) | 178 (32.42) | 216 (39.34) | 51 (9.29) |
| 1. If I have a vaginal delivery, I would choose to receive painless childbirth. (P) | 233 (42.44) | 260 (47.36) | 46 (8.38) | 10 (1.82) |  |
| 1. I am willing to pay extra for painless childbirth services. (P) | 175 (31.88) | 313 (57.01) | 47 (8.56) | 12 (2.19) | 2 (0.36) |
| 1. I believe painless childbirth should be a standard medical service. (P) | 216 (39.34) | 276 (50.27) | 50 (9.11) | 6 (1.09) | 1 (0.18) |
| 1. I believe medical institutions should more actively promote painless childbirth. (P) | 229 (41.71) | 266 (48.45) | 53 (9.65) |  | 1 (0.18) |
| 1. I believe the government should subsidize painless childbirth to improve accessibility. (P) | 327 (59.56) | 203 (36.98) | 17 (3.1) |  | 2 (0.36) |
| 1. I am willing to recommend painless childbirth to other expectant mothers. (P) | 198 (36.07) | 260 (47.36) | 83 (15.12) | 8 (1.46) |  |
| 1. I hope my partner will support my decision to choose painless childbirth. (P) | 273 (49.73) | 250 (45.54) | 25 (4.55) |  | 1 (0.18) |
| 1. The approximate cost of painless childbirth in the local area is 2,600 yuan. Are you able to afford it? (P) | 149 (27.14) | 264 (48.09) | 111 (20.22) | 20 (3.64) | 5 (0.91) |

**Table S5. Practice section of pregnant women in Wuhan regarding painless childbirth**

|  | **N (%)** | | | | |
| --- | --- | --- | --- | --- | --- |
|  | **Always** | **Often** | **Sometimes** | **Rarely** | **Never** |
| 1. Have you ever consulted a doctor about painless childbirth? (P) | 22 (4.01) | 62 (11.29) | 163 (29.69) | 206 (37.52) | 96 (17.49) |
| 1. Have you actively searched for information related to painless childbirth? (P) | 33 (6.01) | 100 (18.21) | 211 (38.43) | 164 (29.87) | 41 (7.47) |
| 1. Have you attended any health education lectures or seminars about painless childbirth? (P) | 8 (1.46) | 33 (6.01) | 80 (14.57) | 218 (39.71) | 210 (38.25) |
| 1. Have you communicated with others (for consultation or recommendation) about painless childbirth? (P) | 23 (4.19) | 71 (12.93) | 218 (39.71) | 177 (32.24) | 60 (10.93) |
| 1. Have you (or would you) shared your thoughts or experiences regarding painless childbirth on social media or online forums? (P) | 13 (2.37) | 34 (6.19) | 110 (20.04) | 183 (33.33) | 209 (38.07) |

# Table S6. Spearman correlation analysis of KAP score and spouse score

|  | Knowledge | Attitude | Practice | Spouse score |
| --- | --- | --- | --- | --- |
| Knowledge | 1 |  |  |  |
| Attitude | 0.543 (P<0.001) | 1 |  |  |
| Practice | 0.528 (P<0.001) | 0.386 (P<0.001) | 1 |  |
| Spouse score | 0.333 (P<0.001) | 0.433 (P<0.001) | 0.373 (P<0.001) | 1 |

# Table S7. Model fit indices of SEM

| **Model Fit Indicators** | **Reference** | **Measured results** |
| --- | --- | --- |
| **CMIN/DF** | 1-3 excellent, 3-5 good | 3.999 |
| **RMSEA** | <0.08 good | 0.074 |
| **IFI** | >0.8 good | 0.900 |
| **TLI** | >0.8 good | 0.888 |
| **CFI** | >0.8 good | 0.900 |

Abbreviations: CMIN/DF, chi-square minimum/degrees of freedom; RMSEA, root mean square error of approximation; IFI, incremental fit index; TLI, Tucker-Lewis index; CFI, comparative fit index

**Table S8. Model path results of KAP scores in SEM analysis**

| **Model Path** | **Estimate** | **Standardized Estimate** | **S.E.** | **C.R.** | **P** |
| --- | --- | --- | --- | --- | --- |
| Spouse scores → Knowledge | 0.030 | 0.326 | 0.004 | 7.269 | <0.001 |
| Knowledge → Attitude | 0.677 | 0.473 | 0.075 | 8.990 | <0.001 |
| Spouse scores → Attitude | 0.039 | 0.299 | 0.005 | 7.184 | <0.001 |
| Attitude → Practice | 0.075 | 0.046 | 0.084 | 0.890 | 0.373 |
| Knowledge → Practice | 1.255 | 0.544 | 0.133 | 9.410 | <0.001 |
| Spouse scores → Practice | 0.039 | 0.186 | 0.009 | 4.352 | <0.001 |
| Knowledge → K1 | 1.000 | 0.699 |  |  |  |
| Knowledge → K2 | 1.610 | 0.782 | 0.094 | 17.132 | <0.001 |
| Knowledge → K3 | 1.430 | 0.749 | 0.087 | 16.444 | <0.001 |
| Knowledge → K4 | 1.564 | 0.787 | 0.091 | 17.227 | <0.001 |
| Knowledge → K5 | 1.402 | 0.779 | 0.082 | 17.053 | <0.001 |
| Knowledge → K6 | 1.536 | 0.787 | 0.089 | 17.219 | <0.001 |
| Knowledge → K7 | 1.582 | 0.788 | 0.092 | 17.239 | <0.001 |
| Knowledge → K9 | 0.758 | 0.473 | 0.072 | 10.516 | <0.001 |
| Knowledge → K10 | 0.926 | 0.514 | 0.081 | 11.423 | <0.001 |
| Attitude → A11 | 1.000 | 0.591 |  |  |  |
| Attitude → A10 | 0.927 | 0.764 | 0.066 | 14.063 | <0.001 |
| Attitude → A9 | 1.265 | 0.854 | 0.084 | 15.078 | <0.001 |
| Attitude → A8 | 0.802 | 0.668 | 0.063 | 12.820 | <0.001 |
| Attitude → A7 | 1.090 | 0.821 | 0.074 | 14.725 | <0.001 |
| Attitude → A6 | 1.170 | 0.846 | 0.078 | 14.989 | <0.001 |
| Attitude → A5 | 1.057 | 0.745 | 0.077 | 13.817 | <0.001 |
| Attitude → A4 | 1.086 | 0.769 | 0.077 | 14.108 | <0.001 |
| Attitude → A3 | 0.932 | 0.489 | 0.093 | 10.057 | <0.001 |
| Attitude → A2 | 1.026 | 0.755 | 0.074 | 13.950 | <0.001 |
| Attitude → A1 | 1.016 | 0.697 | 0.077 | 13.201 | <0.001 |
| Practice → P1 | 1.000 | 0.773 |  |  |  |
| Practice → P2 | 0.960 | 0.767 | 0.054 | 17.852 | <0.001 |
| Practice → P3 | 0.793 | 0.669 | 0.051 | 15.397 | <0.001 |
| Practice → P4 | 0.970 | 0.795 | 0.052 | 18.534 | <0.001 |
| Practice → P5 | 0.834 | 0.652 | 0.056 | 14.980 | <0.001 |
